# Port-site hernia recurrence at previous 5-mm laparoscopic access: case report and review of literature

**DOI:** 10.52054/FVVO.16.2.013

**Published:** 2024-06-28

**Authors:** S Restaino, G Pellecchia, M Arcieri, L Del Pup, G Bogani, L Driul, G Scambia, G Vizzielli

**Affiliations:** Department of Maternal and Child Health, “Santa Maria della Misericordia” University Hospital, Azienda Sanitaria Universitaria Friuli Centrale (ASUFC), 33100 Udine, Italy; Medical Area Department (DAME), University of Udine, 33100 Udine, Italy; Gynaecological Endocrinology and Fertility, University Sanitary Agency Friuli Central (ASUFC), Via Pozzuolo, 330, 33100 Udine, Italy; Gynaecological Oncology Unit, Fondazione IRCCS Istituto Nazionale dei Tumori di Milano, Italy; Women, Children and Public Health Sciences, Fondazione Policlinico Universitario Agostino Gemelli IRCCS, Catholic University of the Sacred Heart School of Medicine, Rome, Italy

**Keywords:** surgeries, laparoscopic, abdominal hernia, case reports, review literature

## Abstract

Port-site hernia (PSH) of less than 10 mm is an exceptionally rare complication of minimally invasive surgery (MIS). To date, there have been no cases in the literature reporting recurrence of PSH from a 5 mm incision. We present the first case of PSH recurrence in a woman who underwent surgery for benign gynaecological pathology via a MIS approach. Her post-operative course was complicated by an episode of symptomatic hernia arising from a 5 mm accessory trocar which was surgically managed. A few months later she re-presented with the same symptoms and had a PSH recurrence of the same port-site. Two corrective surgeries employing different techniques were performed. The first episode was managed laparoscopically using interrupted stitches. On the other hand, the PSH recurrence was managed by placement of a mesh. Ultrasound played a crucial role in diagnostics, especially in the recurrent setting. Due to the complete absence of similar cases in the literature, the decision making around the management of a PSH recurrence from a 5 mm trocar site proved to be challenging. As MIS is the current standard of care, more cases are likely to occur, however despite the increasing number of surgical procedures performed via MIS, no established guidelines for managing such complications have been proposed. Trying to bridge this gap, we present the case report of the first case of PSH recurrence from a 5 mm accessory port and a review of the most significant literature available to date. We finally summarise the reported cases of PSH and the types of surgical repair conducted to highlight the absence of a standard of care.

## Introduction

According to the guidelines of the European Hernia Society an incisional hernia is defined as any gap in the abdominal wall that can be felt or seen during physical examination, regardless of whether there is an extension of the surgical scar area. Although this may occur later, about half of the cases are recorded within the first year after surgery (Ahsan et al., 2023). More than a third of patients experienced pain, discomfort, bowel obstruction, limited mobility, and often a delay in recovery.

Minimally invasive surgery (MIS) has progressed in the era of modern surgery, establishing itself as standard of care. This transition is marked by the emergence of distinct complications compared to those seen in the past. PSH are among them, although they remain rather uncommon (Latyf et al., 2011). PSH most frequently develop in ports with a minimum size of 10 mm (96%), particularly in the umbilical region (82%) (Helgstrand et al., 2011). In the process of describing the incidence of trocar site hernias in gastrointestinal tract surgery, PSH were classified into three groups; early-onset, late-onset and special type (Tonouchi et al., 2004). The early-onset type would occur in the immediate post-operative period, characterised by bowel obstruction due to dehiscence of the anterior and posterior fascial plane and peritoneum. The late- onset type would be due to dehiscence of the anterior and posterior fascial plane, and this may present several months after laparoscopic surgery. Finally, the special type would constitute a dehiscence of the entire abdominal wall with bowel protrusion, omental fat, or other viscera. Currently, there are no specific classifications for PSH arising from MIS pertaining to gynaecological surgery.

We present the first case of PSH recurrence in a previous 5mm port site, after effective laparoscopic repair some months earlier. The literature is yet divided on whether the fascia should be closed in the 5mm trocar site, as a preventive measure. The composition of this paper arose from the unique characteristics of the case, coupled with the lack of unidirectional literature. It aims to offer readers a comprehensive synthesis of the available evidence.

## Case Report

This is a case of a 49-year-old female patient presenting to our gynaecology department in 2022 with a history of heavy periods and fibroids. The patient was offered a total hysterectomy with bilateral salpingectomy and ovarian preservation. Her medical history revealed previous multiple myomectomies by laparotomic approach and a caesarean section. At the time of assessment, the patient was found to have a low anaesthetic risk with a body mass index (BMI) of 29.

She underwent elective laparoscopic surgery and a mini-laparotomic Pfannenstiel incision for intact extraction of the surgical specimen as the uterus was enlarged.

The surgery was performed using a trans- umbilical laparoscopic method with an open technique, employing a 12 mm trocar and three additional blunt-type balloon accessory trocars of 5 mm in diameter in specific locations. As a preventive measure, a 19-Fr Douglas drain was placed with outflow in the right iliac fossa. Later, a mini-laparotomy was performed using a transverse incision of about 7 cm. No intra- operative complications were encountered during the procedure.

A month later, during a gynaecological follow-up, the patient reported abdominal pain in the left iliac tract and minor changes in bowel habits persisting for nearly a month. Upon seeking medical attention for worsening pain, an examination in another hospital revealed a firm and bulge in the left flank. Subsequent tests included a negative abdominal radiograph and a comprehensive gynaecologic ultrasound.

Despite normal healing of the surgical wounds, persistent symptoms led to a colonoscopy, which uncovered a loop of bowel in the left laparocele. Confirmatory ultrasonography indicated a focal discontinuity of the abdominal fibromuscular wall related to the previous MIS approach. Exploratory laparoscopy was therefore performed to correct the PSH. It was performed by an open technique via a trans-umbilical access. Intraoperatively, a 1.5 cm hernial orifice was observed at the site of the previous laparoscopic entry, and careful reduction of the hernia contents was carried out (Figure 1). Fortunately, there were no signs of ischemia or necrosis of the intestinal tract trapped in the hernial port. Due to the size of the hernial orifice, a laparoscopic offset point correction was realised with an intralesional fascial suture using Vicryl interrupted stitches. The patient’s postoperative recovery was uneventful, and she was discharged on the second post-operative day. Three months following the surgical repair, she reported worsening pain in the left iliac fossa, prompting a re-examination. Transabdominal ultrasonography revealed a 2 cm fascial detachment at the site of previous scar in the same place, the left iliac fossa, along with an overlying swelling. Suspecting laparocele recurrence, laparoplasty was performed, reducing the intact hernia sac with a Ventralex- type prosthesis and attaching the tails to the fascia by Prolene 2/0 stitches. The patient was advised to dress the wound with an elastic support bandage and she is currently well and asymptomatic.

**Figure 1:**
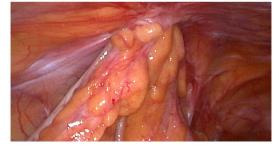
Laparoscopic framework at the first hernia episode on a previous 5 mm trocar. Figure 1A: Entrapment of sigma loop with omental fat.

**Figure 1B:**
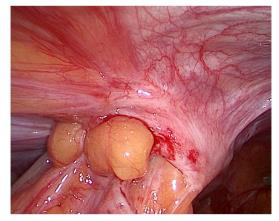
Entrapment of sigma loop.

**Figure 1C:**
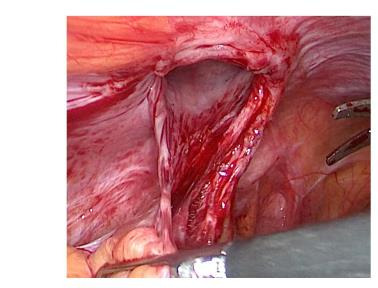
The extent of the fascia defect.

**Figure 1D:**
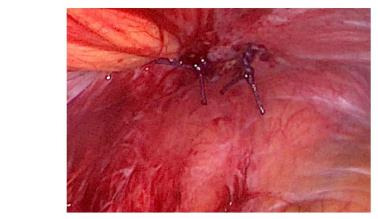
The appearance after correction in detached Vicryl stitches.

## Discussion

PSH was first documented in 1968 within a cohort of patients undergoing laparoscopic gynaecological procedures (Fear, 1968). The prevalence of PSH ranges from 0.38% to 5.4%, with an overall occurrence of 1.7% (Sugrue et al., 2013).

PSH originate from fascial defects at trocar incision sites. Documented risk factors for PSH formation include trocar size, incomplete fascial closure, and organ extraction through the port (Bunting, 2010). Identified risk factors for specified PSH at 5 mm ports involve prolonged trocar manipulation and operative duration surpassing 90 minutes (Reardon et al., 1999). Interestingly, the placement of a drain through a right 5mm port has been proposed as a possible PSH risk factor. In this regard, the practice of externalising the drain through a 5 mm port remains controversial (Bunting, 2010).

A retrospective study conducted by Nezhat et al. (1997) detailing 10 cases of PSH within a cohort of 5,300 women who had undergone laparoscopic gynaecological surgery, half of these cases described occurred at the 5 mm access point. Notably, all cases were observed on the left side rather than in the middle or on the right of the suprapubic site. The authors attributed this observation to increased manipulation with the suction-irrigation catheter and needle holder, both frequently inserted through the left trocar (Nezhat et al., 1997). In our case, both occurrences transpired at the left accessory trocar, seemingly unrelated to drainage insertion, which had been inserted in the contralateral port.

There is also a disparity in the literature regarding the necessity to close a 5 mm trocar defect (Reardon et al., 1999). In the study conducted by Marcinkeviciute et al. (2023) mesh was not employed for hernia repair due to small size of the defect (only 12 mm) and closure with two Prolene sutures was deemed adequate.

The retrospective series by Zhu et al. (2019) reported 9 cases of PSH among more than 55,000 patients, with the hernias occurring predominantly in women over 60-years and following gynaecological procedures performed employing a single incision technique. Among the 9 cases, the 2 occurrences manifested on a 5 mm right trocar, were repaired through open surgery.

Moreover, Schumacher et al. (2003) demonstrated, that if the hernia gap is smaller than 2 cm, suturing the hernia defect without employing mesh is considered safe, and the risk of recurrence is low (4.1%). This appeared to be similar to our approach in the first episode of PSH. In one of the most extensive retrospective case series, 54 cases of hernias on previous laparoscopic trocars were statistically analysed regarding hernia site, type of repair (comparing suture alone versus mesh repair), and recurrence rate. The authors observed that incisional hernias occurred in more than 95% of cases, on ports of at least 10 mm in diameter. In addition, they found the infrequent instances of hernias on a 5 mm access did not necessitate fascia closure during the initial surgery (Lambertz et al., 2017). This assumption is supported by the challenge of fascial closure, which may pose a risk of injuring underlying structures such as intestinal loops or omentum. Importantly, no superiority in hernia recurrence was identified through the comparison of different surgery repair modalities. These findings were corroborated in the latest meta- analysis, which indicated no significant difference in hernia recurrence rates between open repair with mesh and laparoscopic suture approaches (Campanile et al., 2023).

Our case is distinctive within the limited scientific evidence as it represents the initial documented event of the recurrence of PSH at the location of a 5 mm port-site, which is exceedingly rare. In line with the scant recommendations with a low level of evidence, we refrained from using a reconstructive mesh during the initial procedure due to the size of the defect. A mesh was instead placed to repair recurrent PSH.

We attempted to consolidate all known cases of hernias through a 5 mm port-site, along with their corresponding repair techniques (Table I). Notably, none of these cases demonstrates a recurrence. While the probability of developing a post-operative incisional hernia at a site with an incision less than 10 mm is exceedingly low, there are a few examples documented in the literature (Sugrue et al., 2013).

**Table I:** List of cases of 5 mm port-site hernia known in the literature.

Author	Year	Study	Location PSH	Repair
Sugrue et al.	2013	Case report	Two cases of port site hernia on previous 5 mm access in the right iliac fossa	Laparotomic correction by extending the hernial defect to perform appendectomy (hernia complicated by incarcerated appendix).
Yamamoto et al.	2011	Case report	Port-site spigelian hernia on 5 mm access in the right abdominal wall	Peritoneum and fascia closed with a 3/0-polygalactin running stitch
Dulskas et al.	2011	Case report	port site hernia on 5 mm access in the right lateral subcostal margin	the fascial−muscular defect was closed with interrupted Vycril 2/0 sutures.
Reardon et al.	1999	Case report	port site hernia on 5 mm laparoscopic access on the left side of the abdominal wall	The abdominal defect was repaired with interrupted sutures of No. 1 polypropylene
Nezhat et al.	1997	Retrospective study (5300 laparoscopic gynaecological surgeries)	5 cases of port-site hernia on 5 mm access in left iliac fossa	All were treated with laparoscopy, reduction of hernia, and repair of incision
Toub and Campion	1994	Case report and review of literature	One case of 5 mm port-site hernia port-site hernia on 5 mm access in the left lower abdominal quadrant.	Laparoscopy was not attempted, it required a small extension of the wound, which was closed in layers using polydioxanone sulphate suture.
Plaus et al.	1993	Case series	One case of port-site hernia on 5 mm access at the suprapubic site	repaired with 0 Ethibond suture under local anaesthesia

A distinguishing factor from the previously mentioned cases is the time-frame between surgery and hernia development, typically manifesting almost immediately post-operatively. In contrast, our patient exhibited nuanced and inconclusive symptoms that prompted clinical evaluation approximately one month after the surgery. Therefore, the increase in hernial defect size from 5mm during the initial episode to 15 mm is likely imputable to the elapsed time from its development until surgery (Tazaki et al., 2023).

Despite that our case in question presented late, ultrasonography proved to be invaluable to help with the diagnostic uncertainty. The 2019 International Endohernia Society (IEHS) guidelines on the laparoscopic treatment of incisional abdominal hernias delineate the significance of sonography as the gold standard method in the diagnosis of a palpable mass on the abdominal wall from a hernia. The potential role of sonography also remains open and requires further definition in determining the type of correction to be performed, favouring mesh over suture (Chaudhry et al., 2017). This aspect would have played a crucial role in our case at the time of recurrence in deciding the most appropriate surgical correction.

Baz et al. (2019) provide an overview of the ultrasound appearance of various abdominal wall pathologies. Specifically, regarding wall hernias, they highlight certain ultrasound features crucial for the differential diagnosis. These include: determining the location of the wall defect in relation to the inferior epigastric artery as an anatomical landmark for defining a direct hernia; assessing the location of the defect in relation to the umbilicus and identifying the type of herniated content for a ventral hernia; locating the defect on a previous surgical scar for an incisional hernia; and recognising the same location of a previous surgical correction for a recurrent hernia (Baz et al., 2019).

With regards to PSH prevention, the majority of studies do not provide specific recommendations for the fascial closure of 5 mm trocar defects or they leave the choice to the surgeon on a case- by-case basis. Laparoscopic techniques may be contemplated for mending smaller defects. It may be advised to close trocar sites with fascial defects of 10 mm or more, encompassing the peritoneum.

Some studies even suggest that with a paramedian location and use of blunt-type trocars, fascial closure is not deemed crucial not only for 5 mm but also for 10 mm and 12 mm incisions. In fact, many studies do not advocate for fascial closure in the case of 5 mm trocar defects (Yamamoto et al., 2011), as the blunt trocars utilised for 5 mm trocar sites split the muscles instead of cutting them, thereby reducing the area of the fascial defect.

Several additional preventive measures include using smaller diameter trocars whenever possible, avoiding excessive manipulation of trocars, employing the Z-incision technique for trocar insertion, ensuring direct visual haemostasis, and removing them carefully.

An alternative option to consider for preventing incisional trocar site hernia is the utilisation of Deschamps’ needle. In their prospective series, Di Lorenzo et al. (2002) employed this simple and cost-effective device to perform and close 5 and 10 mm incisions, observing no wound dehiscence or hernias at these sites. While this surgical technique provides valuable insights, it is not widely used or standardised. In this regard, Dulskas et al. (2011) recommended it for the closure of laparoscopic accesses larger than 10 mm in diameter.

Another noteworthy aspect of discussion about prevention is the selection of surgical instrumentation, particularly laparoscopic trocars. A recent comprehensive systematic review comparing single-use and reusable laparoscopic trocars revealed substantial heterogeneity and a scarcity of data on the theoretical advantages of single-use trocars in terms of efficacy, safety, sterility, ease of use, and improved patient outcomes. (Siu et al., 2017). The study by Lau et al. (2002) highlighted potential drawbacks of reusable metallic trocars, including peri-cannular air leaks and, more importantly, sliding movements of trocars during insertion and withdrawal. In contrast, disposable trocars, as used in our patient, offer the advantage of being equipped with an anchoring device to the abdominal wall, allowing immediate fixation after insertion to prevent dislocation and risky movements to the fascia. In a pig study comparing 5 types of disposable trocars, the authors found that radially dilating trocars required the highest insertion force compared with the hybrid dilating and plastic blade trocar system, but the removal force was instead equivalent for all trocars. The size of fascial defects, both in terms of functional and anatomical aspects, was smallest with the hybrid and radially dilating trocar systems. The authors concluded that fascial defects created by as much as 10 and 12 mm hybrid dilating trocars may not necessitate closure, emphasising the importance of individualising the choice based on the patient’s nutritional state and comorbidities (Shafer et al., 2006).

In this case, on questioning the patient revealed a family history of post-surgical laparocele, with a similar complication occurring in a sibling. This might suggest other underlying causes, such as defects in the collagen matrix. Although lacking comparative case histories, we can only speculate on potential underlying factors at this juncture. The literature contains experimental and non-standardised data supporting collagen turnover protein deficiency in the etiopathogenesis of incisional hernias. In a prospective series, Durukan et al. (2022) found that tissue inhibitor metalloproteinases type-1 levels were significantly lower, while type-2 levels were significantly higher in inguinal and incisional hernia groups compared to the control group. Further verification through randomised controlled trials on larger patient cohorts may substantiate the utility of TIMP levels in clinical practice (Durukan et al., 2022). Despite advancements, the aetiology and biology of incisional hernias after laparotomic surgery remain extensively studied. Establishing a standard requires demonstrating that the same pathogenetic biology also underlies incisional hernias from previous laparoscopic trocar procedures. A recent systematic review of the literature revealed several extracellular matrix alterations as potential biomarkers for abdominal hernias (Pilkington et al., 2021). However, there is an absence of specific targeting for port site hernia in current research.

## Conclusions

There are no unequivocal recommendations regarding the optimal repair method for port-site hernias at the site of a previous 5 mm access. This lack of clarity justifies our decision to adopt a laparoscopic restorative approach during the initial episode and to incorporate mesh at the time of recurrence. Further research is warranted on several fronts, ranging from the biology and pathogenesis of hernias arising from previous laparoscopic trocars to the desirable standardised treatment for smaller calibre hernias occurring at 5 mm access points, and their associated risk factors.
